# Impact of Dose and Sensitivity Heterogeneity on TCP

**DOI:** 10.1155/2014/182935

**Published:** 2014-05-12

**Authors:** Kristin Wiklund, Iuliana Toma-Dasu, Bengt K. Lind

**Affiliations:** ^1^Medical Radiation Physics, Stockholm University, 106 91 Stockholm, Sweden; ^2^Department of Oncology and Pathology, Karolinska Institutet, P.O. Box 260, 171 76 Stockholm, Sweden

## Abstract

This present paper presents an analytical description and numerical simulations of the influence of macroscopic intercell dose variations and intercell sensitivity variations on the probability of controlling the tumour. Computer simulations of tumour control probability accounting for heterogeneity in dose and radiation sensitivity were performed. An analytical expression for tumor control probability accounting for heterogeneity in sensitivity was also proposed and validated against simulations. The results show good agreement between numerical simulations and the calculated TCP using the proposed analytical expression for the case of a heterogeneous dose and sensitivity distributions. When the intratumour variations of dose and sensitivity are taken into account, the total dose required for achieving the same level of control as for the case of homogeneous distribution is only slightly higher, the influence of the variations in the two factors taken into account being additive. The results of this study show that the interplay between cell or tumour variation in the sensitivity to radiation and the inherent heterogeneity in dose distribution is highly complex and therefore should be taken into account when predicting the outcome of a given treatment in terms of tumor control probability.

## 1. Introduction

Modern advanced radiation therapy makes use of radiobiological models for tumor control probability (TCP) for predicting the outcome and optimizing the treatment as well as for treatment evaluation. The most widely used model for the calculation of the probability of eradicating the tumor is based on the linear-quadratic (LQ) model for cell survival in conjunction with Poisson statistics describing the distribution of the clonogenic cells surviving at the end of the treatment. The standard expression for TCP assumes that all the cells in the irradiated volume have the same intrinsic sensitivity to radiation and the same sensitivity to changes in the fractionation. Furthermore, the model also assumes that all the cells receive the same dose. Refined versions of the model proposed solutions for accounting for heterogeneities in the dose distribution by dividing the tumor volume into subvolumes down to voxel size in which the dose and the radiation sensitivity were assumed to be constant [1–3]. The influence of heterogeneity in the intrinsic sensitivity of the cells to radiation within the tumor or from one tumor to another or of the number of clonogenic cells between tumors on the overall outcome was also theoretically explored by several authors [3–12]. Similar studies have been performed with computer simulations by Harting et al. [13].

However, to the best of our knowledge, the combined influence on TCP from both the deterministic and the intrinsic stochastic heterogeneity in dose delivery and the variation of the sensitivity of the cells to radiation on TCP has not yet been fully explored.

It is therefore the aim of the current paper to present a comprehensive analysis of the TCP, after irradiation with heterogeneous dose distributions in the presence of differences between cells with respect to their radiation sensitivity, in fractionated radiation therapy. The analysis is based on the LQ description of the cell survival and includes the effect of cell proliferation. The study explores the effect on TCP when exposing cell populations with various irradiation patterns under different assumptions of the cells sensitivity or with intertumor variation in sensitivity.

Closed form expressions for the tumor control probability that take into account sensitivity variation within a tumor has, to the best of our knowledge, not been published. Therefore, an analytical formalism for calculations of TCP has been proposed, in order to determine and explain the dose response in presence of heterogeneity either in dose to the cells or sensitivity of the cells within a tumor.

## 2. Materials and Methods

### 2.1. Computer Simulation of TCP Accounting for Heterogeneity in Dose and Sensitivity

The probability of controlling the tumors under the assumption that the LQ model describes the response of individual cells to radiation and that a Poisson distribution describes the number of clonogens *N* left after receiving *n* fractions of a dose *d* is given by

(1) $$\begin{array}{c} P=\exp\left(-N_{0}s^{n}\left(d\right)\right)=\exp\left(-\lambda \right), \end{array}$$

where *P* denotes the probability of tumors control, referred to in the text as TCP, *N*_0_ is the initial number of clonogens, and *s* is the probability of survival in each fraction. Consequently *N* ~ *Po*(*λ*) with *EN* = *λ* and (1) implicitly assumes that every clonogen has to be eradicated in order to control the tumor.

In fractionated radiation therapy the cells that survive irradiation will get the chance to proliferate. In order to account for proliferation one could introduce a proliferation factor depending on the clonogen doubling time, *T*_*D*_, and overall treatment time, *t*, *R* = exp⁡((ln⁡2)*t*/*T*_*D*_) so that (1) becomes

(2) $$\begin{array}{c} P=\exp\left(-\lambda R\right)=\exp\left(-\lambda_{R}\right), \end{array}$$

which unfortunately results in that the number of clonogens, when proliferation is present, is no longer Possion distributed (*N*_*R*_≁*Po*(*λ*)) since

(3) $$\begin{array}{c} EN_{R}=\lambda_{R}=\lambda R\neq \text{V}N_{R}=\lambda R^{2}. \end{array}$$

Several studies have questioned the validity of using Poisson statistics with a simple multiplicative factor (see (2)) to account for the proliferation for describing the tumor control in case of highly proliferating tumors raising a warning against its use [14–16] and proposing improved or new models for overcoming the problem [17–19]. Hanin et al. [20] gave the exact distribution for the number of clonogens. The recursive algorithm proposed by Deasy [17] could be used for determining the probability of an initial clonogen to give rise to one or more surviving clonogens at the end of the treatment when the dose and sensitivity are homogeneously distributed over the tumor volume.

According to Deasy [17], the probability of controlling the tumor is given by

(4) $$\begin{array}{c} P={\left(1-G_{1}\right)}^{N_{0}}, \end{array}$$

where *G*_1_ is the probability of an initial clonogen giving rise to at least one clonogen surviving at the end of treatment. Equation (4) would thus give the tumor control probability accounting for cell proliferation, assuming that the dose is delivered homogeneously and all the cells have the same radiation sensitivity which remains constant during the duration of the treatment. This formula is derived with the assumption that each clonogen divides only once between the fractions.

In the present paper the probability of controlling the tumor was calculated as the average TCP, resulting from the simulation of the irradiation of a large number of tumors having the same average growth kinetics and radiation sensitivity and containing the same number of initial clonogens. The simulations are based on the assumption that all clonogens have to be eradicated in order to control the tumor. The cell survival is sampled from a Bernoulli distribution where the probability for a given cell to survive is described by the LQ model:

(5) $$\begin{array}{c} S\left(d\right)=\exp\left(-\alpha d-\beta d^{2}\right), \end{array}$$

where *d* is the dose per fraction and *α* and *β* are the linear and quadratic parameters, respectively.

If the cells potential doubling time is *T*_pot_, the probability for a cell to divide once in between fractions is given by

(6) $$\begin{array}{c} p_{\mathrm{div}}=2^{t/T_{\text{pot}}}-1, \end{array}$$

with the assumption of a fractionation interval, *t*, of one day and with the constraint *T*_pot_ ≥ *t*. For a realistic simulation of the tumor growth, the potential doubling time, *T*_pot_, could be replaced by *T*_*D*_, the effective doubling time which takes into account the cell loss. The probability that the surviving cells will undergo one cell division between two consecutive fractions is also sampled from a Bernoulli distribution with the probability of a cell to divide given by (6). The assumption of only one cell division between two consecutive fractions with the probability of division given by (6) will cause an error due to neglecting the probability for a cell to divide more than once. This error can be estimated by 1 − *p*_0_ − *p*_1_, where *p*_*k*_ = *λ* ^*k*^ *e* ^−*λ*^/*k*!. For a *T*_*D*_ of 2 days, used in the present calculation, the error will be around 5%. The influence of the heterogeneity in dose on the control of the tumor was calculated by performing numerical simulations, in which the dose was randomly sampled from a normal distribution with a specified mean dose and standard deviation as described in [21]. The coefficient of variation was set to 20%. A 20% variation from the mean dose delivered to the cells in the tumor could represent either the worst case for the situation when the tumor is intended to be irradiated with a homogeneous dose but the set-up was incorrect or the movement of the tumor was not properly anticipated, or it could represent an intentional variation in dose as in an integrated boost or a dose-painting approach. It could also be regarded as a theoretical test whether there could be benefits from treating tumors with intentionally heterogeneous dose distributions.

In order to account for variations in the sensitivity of the cells to radiation, one could assume that the individual radiation sensitivity of the cells in a population follows a given log-normal distribution [12]. The heterogeneity in sensitivity could then be simulated by sampling the values from the assumed distribution. If the sensitivity of the cells to radiation is defined with the help of the parameters of the LQ model, there could be several options to consider when performing the sampling of the parameters in order to mimic a heterogeneous tumor with respect to sensitivity. Thus, two different options were explored in this paper for the simulation of intratumor heterogeneity of the cell sensitivity:

1. the *α* value was sampled at each fraction from a log-normal distribution for all cells in the tumor while keeping the *β* value constant, which also results in a variable cellular *α*/*β*;
2. the *α* value was sampled once from a log-normal distribution whenever a cell is born while keeping the *β* value constant which results in a variable cellular *α*/*β*. The distribution is kept the same throughout the whole treatment.

### 2.2. Analytical Description of TCP Accounting for Heterogeneity in Dose and Sensitivity

An analytical expression for TCP, based on a binomial expression with the assumption that every clonogenic cell has to be eradicated in order to control the tumor, that takes into account the heterogeneity in dose at cellular level was presented in a previous paper [21]. According to that, a Taylor expansion of the expression for the tumor control probability around the mean dose per fraction to the cells would lead to

(7) P≈P(D)exp⁡(nN02σc2[s′2s2n−2(1−sn)2+s′′sn−11−sn])≈P(D){1−nN02σc2[s′2s2n−2(1−sn)2+s′′sn−11−sn]},

where *σ*_*c*_ is the standard deviation of the dose to a given cell, *P*(*D*) is the TCP for a homogeneous total dose *D* delivered in *n* fractions, and *s*, *s*′, and *s*′′ are the functions describing cell survival and first and second derivative of the cell survival as function of dose, respectively. If the LQ model is used to describe the cell survival, the derivatives are

(8) s′(d)=(−α−2βd)exp⁡(−αd−βd2)=(−α−2βd)ss′′(d)=(−2β)exp⁡(−αd−βd2) +(−α−2βd)2exp⁡(−αd−βd2)=(−2β+(−α−2βd)2)s.

Inserting these expressions in (7), the resulting expression becomes

(9) P≈P(D){1−nN02σc2[(α+2βd)2s2n(1−sn)2          +(−2β+((α+2βd)2))sn1−sn]}.

For the particular case when the parameter *β* is zero, the derivatives become

(10) $$\begin{array}{c} s^{\prime }\left(d\right)=-\alpha s\\ s^{\prime \prime }\left(d\right)=\alpha^{2}s. \end{array}$$

The resulting expression for TCP would thus become

(11) $$\begin{array}{c} P\approx P\left(D\right)\left\{1-\frac{nN_{0}\alpha^{2}}{2}\sigma_{c}^{2}\left[\frac{s^{2n}}{{\left(1-s^{n}\right)}^{2}}+\frac{s^{n}}{1-s^{n}}\right]\right\}. \end{array}$$

If the sensitivity to radiation is heterogeneous, a similar formalism for determining the tumor control probability could be applied by performing a Taylor expansion of the expression for the tumor control probability around the mean *α* value of the cells in the tumor:

(12) $$\begin{array}{c} P\approx P\left(\alpha \right)\left\{1-\frac{nN_{0}}{2}\sigma_{\alpha }^{2}\left[\frac{C^{2}s^{2n}}{{\left(1-s^{n}\right)}^{2}}+\frac{C^{2}s^{n}}{1-s^{n}}\right]\right\}, \end{array}$$

where *σ*_*α*_ is the standard deviation of the *α* value for a cell, *C* the inner derivative of the expression for survival, and *P*(*α*) is the TCP in case of a constant *α* value. Using the LQ model and treating the *β* value as a constant, the derivatives explicitly read

(13) $$\begin{array}{c} s^{\prime }\left(d\right)=-ds,\\ s^{\prime \prime }\left(d\right)=d^{2}s. \end{array}$$

The resulting expression becomes

(14) $$\begin{array}{c} P\approx P\left(\alpha \right)\left\{1-\frac{nN_{0}d^{2}}{2}\sigma_{\alpha }^{2}\left[\frac{s^{2n}}{{\left(1-s^{n}\right)}^{2}}+\frac{s^{n}}{1-s^{n}}\right]\right\}. \end{array}$$

In order to investigate how the effect of dose heterogeneity varies with the *α*/*β* ratio and *SF*_2_, that is, whether the effect on TCP is positive or negative, one can rewrite (7) as

(15) $$\begin{array}{c} P\approx P\left(D\right)\left\{1-\frac{nN_{0}}{2}\sigma_{\text{c}}^{2}f\left(\frac{\alpha }{\beta },SF_{2}\right)\right\}. \end{array}$$

A negative effect on TCP from the heterogeneity in dose means that the control probability achieved when the dose is uniformly delivered to all cells is higher than the TCP resulting from an irradiation with a heterogeneous dose distribution with the same mean value as the dose delivered uniformly. This situation corresponds to the case when the quantity in the square brackets in (7), denoted by *f*(*α*/*β*, *SF*_2_) in (15), is positive. A positive effect of the dose heterogeneity on TCP means that the actual control probability of the tumor is higher when the dose is delivered nonuniformly to the cells than when the dose is the same to all cells. This is achieved when *f*(*α*/*β*, *SF*_2_) in (15) is negative.

## 3. Results and Discussions

**Figure 1:**
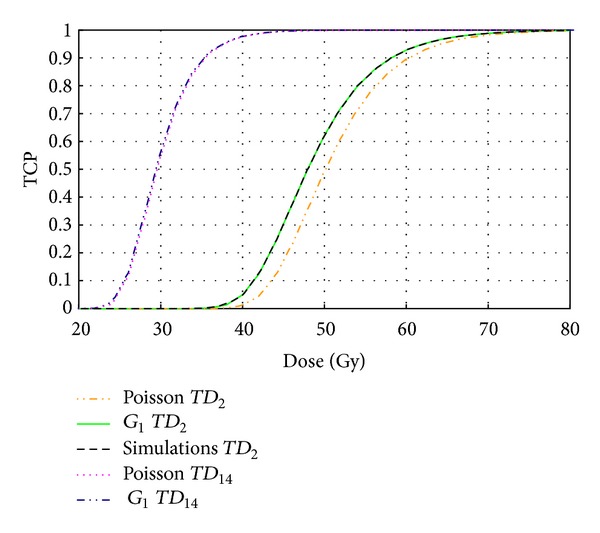
Comparison between dose response calculated with the Poisson expression where the repopulation correction is made by the multiplicative factor *R*, calculations by using expression (4), and numerical simulations, for two effective doubling times, *T*_*D*_ = 2 days and *T*_*D*_ = 14 days. Probability of survival is 0.50 and the dose per fraction is 2 Gy.

Several approaches for determining the probability of tumor control have been presented in the previous section: the Poisson-LQ model including the effect of proliferation, (2), the improved model based on the recursive algorithm, (4), and the simulation of the probability of cell survival and proliferation.  Figure 1 shows a comparison of the dose response curves giving the TCP calculated with these different methods as a function of the total dose delivered in 2 Gy per fraction for a rapidly proliferating tumor (*T*_*D*_ = 2 days) and a slowly proliferating tumor (*T*_*D*_ = 14 days) with the same intrinsic sensitivity (*SF*_2_ = 0.5). As expected, for short cell doubling time, the Poisson-LQ model, including proliferation, overestimates the number of surviving cells and consequently underestimates the TCP. The corrected version of the model proposed by [17] agrees perfectly with the full computer simulation. For slowly proliferating tumors, the number of surviving tumor clonogens at the end of the treatment is almost Poisson distributed, and, hence, the dose response curves calculated with the original Poisson-LQ model and with the improved version almost coincide.

The results in Figure 1 could thus be interpreted as a validation of the simulations against the corrected version of the Poisson-LQ model for the case of a homogeneous dose and sensitivity distribution. Once the validation was performed, the simulation approach was used to investigate the influence of the heterogeneity in dose and intratumor radiation sensitivity on TCP.

**Figure 2:**
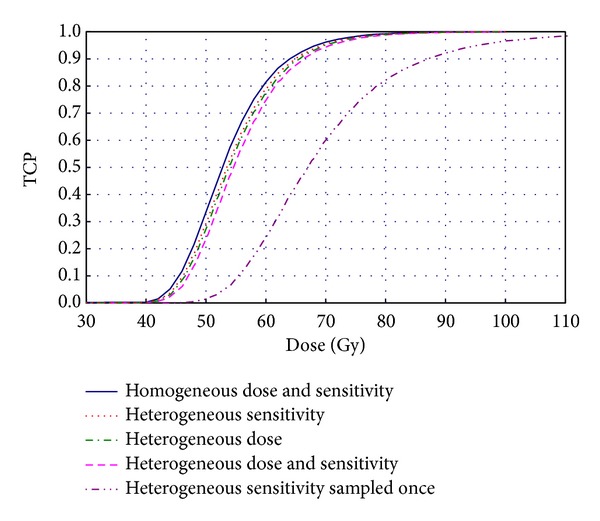
Intratumor variation in dose and sensitivity for a tissue with *α*/*β* = 10 Gy and *T*_*D*_ = 2 days. The coefficient of variance is in all cases 20%.

Figure 2 shows the dose response curves for a rapidly proliferating tumor containing an initial number of 10^4^ cells before the start of the treatment for various scenarios regarding the distribution of the parameters influencing the TCP. The dose response curve for the case of homogeneous distributions of the total dose delivered in 2 Gy per fraction and the same intrinsic sensitivity described as *SF*_2_ = 0.5 and sensitivity to fractionation *α*/*β* = 10 Gy for all the cells is also indicated (solid dark blue curve). One could observe in Figure 2 that if there is a variation of 20% in the radiation sensitivity of the cells within the tumor resulting from a distribution of alpha values around the mean value, corresponding to *SF*_2_ = 0.5 and *α*/*β* = 10 Gy, while the *β* term is kept constant (option 1 for sensitivity sampling in Section 2), the dose required to achieve a certain value of TCP is only slightly higher than the dose corresponding to the case when both the sensitivity and the dose were the same for all the cells (dashed red curve). The situation described by option 1 could reflect the case of a tumor with dynamic hypoxia under the assumption that the hypoxic fraction remains constant between the fractions. The trend of increasing the dose to achieve the same level of control is kept for the case when the cells had the same response per unit dose, hence the same sensitivity, but the dose distribution was characterized by a coefficient of variation (CV) = 20% around the mean (dashed green curve). The influence on the control probability, for this specific simulated case, from the heterogeneity in dose and the variation in sensitivity from cell to cell within the tumor, results in a further slight increase in the dose required for achieving the same level of control (dashed pink curve) as the effects of the two distributions are adding up.

In order to quantify the effects on TCP from two or more heterogeneities, one can define a coefficient of synergy:

(16) $$\begin{array}{c} \frac{\Delta P_{\alpha }+\Delta P_{\text{dose}}}{P_{\text{tot}}-P_{0}}, \end{array}$$

where Δ*P*_*α*_ and Δ*P*_dose_ are the effect on TCP from cell to cell variations in sensitivity and dose, respectively, *P*_tot_ is the combined effect from dose and sensitivity variations, and *P*_0_ is the TCP without any heterogeneities. If the coefficient is greater than one, the two variations give a synergistic effect, while a coefficient less than one gives a combined effect that is lower than the sum of the two separate variations. For the specific case of *SF*_2_ = 0.5 and *α*/*β* = 10  Gy, used in the present work, a coefficient close to one is found for intratumor sensitivity variation combined with variations of the dose to the cells. If the *α* values that describe the sensitivity of the cells are sampled only once, at the beginning of the irradiation (option 2 for sensitivity sampling in Section 2), the overall radiation resistance of the tumor relative to the initial value at the start of the treatment will gradually increase during the course of fractionated treatment due to the preferential killing of the sensitive cells. This relative decrease in radiation sensitivity is reflected by the dashed purple curve in Figure 2 which has a considerable displacement on the dose axis in comparison to all other curves. This situation could correspond to a tumor with a chronically hypoxic region that does not reoxygenate during the course of the treatment. The first fractions of radiation would preferentially kill the well oxygenated sensitive cells, while the poorly oxygenated surviving cells would require higher doses to be eradicated, hence the shift of the dose response curve towards higher values for the dose. A similar trend was observed by [22] for the case of varying radiation sensitivity due to the presence of hypoxia. It could therefore be noted that the coupled effect of heterogeneity in dose and sensitivity leads to only moderate increase in the total dose to achieve the same tumor control as in the case of heterogeneous dose distribution and homogeneous sensitivity if the treatment is delivered in a relatively large number of fractions, that is, not in a hypofractionated manner.

**Figure 3:**
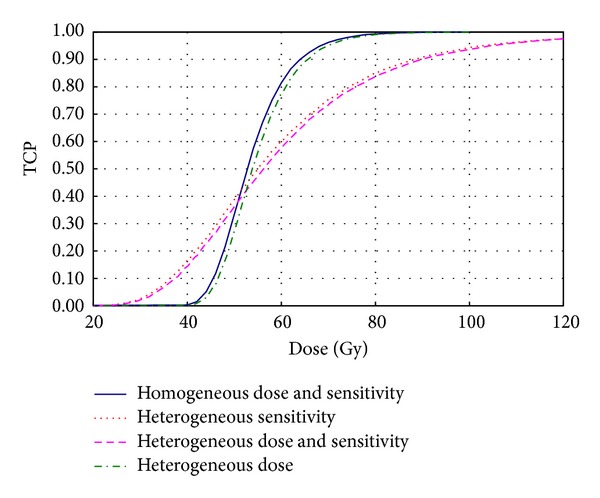
Interpatient variation in sensitivity, intratumor variation in dose, and a combination of those two for a tissue with *α*/*β* = 10 Gy and *T*_*D*_ = 2 days. The coefficient of variance is in all cases 20%.

For a given type of tumors with respect to histopathology and stage, the shape, position, and slope of clinically derived dose response curves depend on the actual heterogeneity of the parameters describing the response of the individual tumors.  Figure 3 shows the influence of interpatient variability in sensitivity for typical tumors (*SF*_2_ = 0.5, *α*/*β* = 10 Gy) containing 10^4^ cells before the start of the treatment together with the curve corresponding to the idealized case of uniform sensitivity among patients irradiated with homogeneous dose distributions (blue solid curve). If the homogeneity of the dose distribution was altered and the dose was sampled from a distribution centered on the value of the dose considered in the homogeneous irradiation with a CV of 20%, the curve only translates to slightly higher dose values (dashed green curve). As expected, if the sensitivity was changed by sampling the values of the *α* parameter once for each tumor while keeping the *β* value constant, in order to simulate the interpatient heterogeneity in sensitivity, the well known reduction of the steepness of the curve was seen (dashed red curve). The effect is even more pronounced when the heterogeneity in sensitivity is combined with heterogeneity in dose (dashed pink curve). The coefficient of synergy is for this case less than one, implying that the effects from the combination of heterogeneity in interpatient sensitivity and in the dose to the cells gives less effect than just adding the effects from respective case separately. The normalized dose response gradient of the curves changed from 3.1 to 1.2. These results confirm the findings in previous studies showing that only distributed parameters describing the sensitivity of the cells and/or the initial number of clonogenic cells could explain the clinically observed steepness of the dose response curves [12, 23]. The present study however has not accounted for variations of *N*_0_ between patients due to the complexity of the analysis when all the combined contributions of distributed parameters would be considered. The results of the simulations are valid only for schedules that allow a low number of cell divisions between consecutive fractions. This may be best illustrated by the case of conventionally fractionated regimes with daily fractions. Gaps of several days and proliferation during them might however lead to some differences from the predictions of this model as indicated in Section 2. This might be the case of some hypofractionated regimes that may depart from daily treatment regimes.

**Figure 4:**
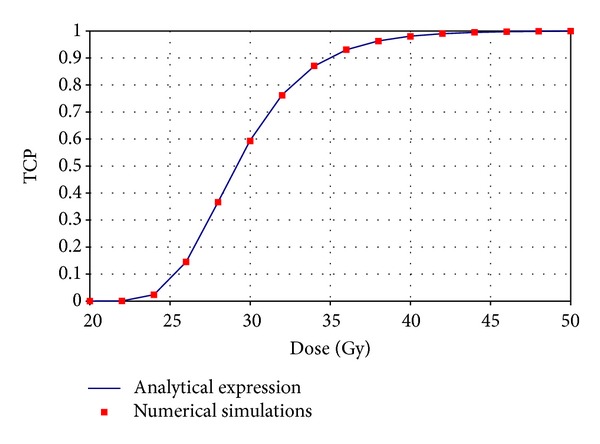
Comparison of the analytical expression for sensitivity variations given by (14) and numerical simulations.

The analytical approach used for describing the influence of the heterogeneity in sensitivity on the control probability was validated by comparing the TCP calculated using (14) with full simulations. The results showing good agreement between the analytical expression and simulations can be seen in Figure 4 for a typical tumor (*SF*_2_ = 0.5, *α*/*β* = 10 Gy, coefficient of variance of the *α* parameter CV = 20%) uniformly irradiated with the indicated total dose delivered in 2 Gy per fraction. The calculated values are perfectly overlapping on the simulated curve proving that the theoretical approach could be used as a fast tool for determining the decrease in TCP if the dose is uniform but the sensitivity of the cells vary within the tumor. A similar validation of the analytical expression for the influence on tumor control of dose variation given by (7) was previously performed against simulations by [21].

**Figure 5:**
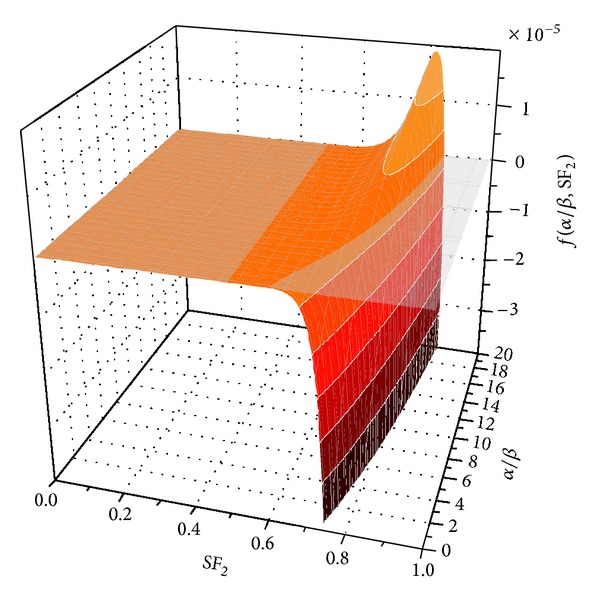
*f*(*α*/*β*, *SF*_2_) plotted for 25 fractions and 2 Gy per fraction.

The influence of the heterogeneity in dose on the control probability described by (15) was investigated by plotting *f*(*α*/*β*, *SF*_2_) as a function of the two parameters. The results are shown in Figure 5. One could observe that for rather resistant tumors (*SF*_2_ ranging from about 0.7 to 1) the effect of the heterogeneity in sensitivity is positive with respect to TCP due to a *f*(*α*/*β*, *SF*_2_) < 0. A positive effect means, in this context, that the actual control of the tumor is higher when the dose is delivered nonuniformly to the cells than when the dose is the same to all cells. However, for a narrow interval of combinations of the values for *SF*_2_ corresponding to rather resistant tumors with *SF*_2_ between approximately 0.6 and 0.8 and higher *α*/*β* than about 5 Gy, *f*(*α*/*β*, *SF*_2_) > 0, resulting in a negative effect on TCP of the heterogeneity in dose meaning that the control probability achieved when the dose is uniformly delivered to all cells is higher than the TCP resulting from an irradiation with a heterogeneous dose distribution with the same mean value as the dose delivered uniformly. The absolute value of the effect is amplified if the number of cells and the coefficient of variation (CV) increase. The complex dependence of the absolute effect on the number of fractions was analyzed by [21] by minimizing the expression inside the square brackets in (7). If a similar analysis would be performed for the expression inside the square brackets in (14), one should notice that the expression does not depend on *α*/*β* and that none of the terms inside the brackets can change sign. This would lead to a much simpler negative expression which is *α*/*β* independent, so the effect of the heterogeneity in the *α* parameter while keeping *β* constant would always be negative, that is, a decrease in the TCP in comparison to the case when *α* is the same for all the cells.

Modern advanced radiation therapy implies the use of functional and molecular imaging for mapping the tumor in terms in radiation sensitivity and nonhomogeneous dose distributions as in dose-painting approaches. The findings of this study have the potential to contribute to the better understanding of the interplay between variations in radiation sensitivity between cells within the tumor as determined based on functional and molecular imaging and the delivered dose distribution, which is currently one of the most exciting challenges in the clinical practice.

## 4. Conclusions

The results of the modeling and the simulations performed in this study show that the interplay between cell or tumor variation in the sensitivity to radiation and the inherent heterogeneity in dose distribution should be taken into account when predicting the outcome of a given treatment in terms of control probability. Furthermore, the findings of this paper should also be taken into account when attempting to determine the parameters describing the radiation sensitivity based on clinically derived dose response curves.
